# Supplementary material for: MetaQTL: a package of new computational methods for the meta-analysis of QTL mapping experiments
Source: BMC Bioinformatics. 2007 Feb 8;8:49. doi: 10.1186/1471-2105-8-49 (PMC1808479; doi:10.1186/1471-2105-8-49)
Supplement: Additional File 7 — MetaQTL Package : jar file and tutorial. This Zip archive contains both the MetaQTL JAR file and the files of the tutorial. [file 1471-2105-8-49-S7.zip › org.inra.metaqtl/doc/org/metaqtl/algo/MetaAlgorithm.html]

MetaAlgorithm


|  |  |  |
| --- | --- | --- |
| **Overview** \| **Package** \| **Class** \| **Use** \| **Tree** \| **Deprecated** \| **Index** \| **Help** | |  |
| **PREV CLASS**   **NEXT CLASS** | **FRAMES**    **NO FRAMES**     **All Classes** |  |
| SUMMARY: NESTED \| FIELD \| CONSTR \| METHOD | DETAIL: FIELD \| CONSTR \| METHOD |  |


---


## org.metaqtl.algo Class MetaAlgorithm

```
java.lang.Object
  org.metaqtl.algo.MetaAlgorithm
```

**All Implemented Interfaces:**: IMetaAlgorithm

**Direct Known Subclasses:**: ClustAlgorithm, MetaMapAlgorithm, QtlProjAlgorithm

---

``` public abstract class MetaAlgorithm extends java.lang.Object implements IMetaAlgorithm ```

---

| **Field Summary** | |
| --- | --- |
| `protected  java.io.PrintStream` | `logger`             The logger. |
| `protected  boolean` | `loggerUp`             The logger status. |
| `protected  int` | `workAmount`             The amount of work to do. |
| `protected  int` | `workProgress`             The amount of work done. |


**Constructor Summary**

`MetaAlgorithm()`


| **Method Summary** | |
| --- | --- |
| `void` | `disableLogger()` |
| `protected  java.io.PrintStream` | `getLogger()`             Returns the logger for this algorithm as a `PrintStream`. |
| `int` | `getWorkAmount()`             Returns the amount of the work to do. |
| `int` | `getWorkProgress()`             Returns the amount of work which have already been done. |
| `boolean` | `isLoggerEnable()` |
| `void` | `setLogger(java.io.OutputStream stream)`             Give a `OutputStream` to the algorithm in order to write out the log info. |
| `void` | `setLogger(java.io.Writer writer)`             Give a `Writer` to the algorithm in order to write out the log info. |

**Methods inherited from class java.lang.Object**

`clone, equals, finalize, getClass, hashCode, notify, notifyAll, toString, wait, wait, wait`

**Methods inherited from interface org.metaqtl.IMetaAlgorithm**

`run`

**Field Detail**

### workAmount

```
protected int workAmount
```

:   The amount of work to do.

---


### workProgress

```
protected int workProgress
```

:   The amount of work done.

---


### logger

```
protected java.io.PrintStream logger
```

:   The logger.

---


### loggerUp

```
protected boolean loggerUp
```

:   The logger status.


**Constructor Detail**

### MetaAlgorithm

```
public MetaAlgorithm()
```


**Method Detail**

### getWorkAmount

```
public int getWorkAmount()
```

:   **Description copied from interface: `IMetaAlgorithm`**
:   Returns the amount of the work to do.

    :   **Specified by:**: `getWorkAmount` in interface `IMetaAlgorithm`
    :   **Returns:**

---


### getWorkProgress

```
public int getWorkProgress()
```

:   **Description copied from interface: `IMetaAlgorithm`**
:   Returns the amount of work which have already
    been done.

    :   **Specified by:**: `getWorkProgress` in interface `IMetaAlgorithm`
    :   **Returns:**

---


### setLogger

```
public void setLogger(java.io.Writer writer)
```

:   **Description copied from interface: `IMetaAlgorithm`**
:   Give a `Writer` to the algorithm
    in order to write out the log info. The user
    is responsible for closing the stream.

    :   **Specified by:**: `setLogger` in interface `IMetaAlgorithm`

---


### setLogger

```
public void setLogger(java.io.OutputStream stream)
```

:   **Description copied from interface: `IMetaAlgorithm`**
:   Give a `OutputStream` to the algorithm
    in order to write out the log info. The user
    is responsible for closing the stream.

    :   **Specified by:**: `setLogger` in interface `IMetaAlgorithm`

---


### getLogger

```
protected java.io.PrintStream getLogger()
```

:   Returns the logger for this algorithm as a
    `PrintStream`. If any logger has
    been previously defined then the method returns
    the default logger, i.e `System.err`

    :   **Returns:**

---


### disableLogger

```
public void disableLogger()
```

---


### isLoggerEnable

```
public boolean isLoggerEnable()
```


---


|  |  |  |
| --- | --- | --- |
| **Overview** \| **Package** \| **Class** \| **Use** \| **Tree** \| **Deprecated** \| **Index** \| **Help** | |  |
| **PREV CLASS**   **NEXT CLASS** | **FRAMES**    **NO FRAMES**     **All Classes** |  |
| SUMMARY: NESTED \| FIELD \| CONSTR \| METHOD | DETAIL: FIELD \| CONSTR \| METHOD |  |


---
